# Microstructure Evolution and Mechanical Properties of Grinding Metamorphic for 8Cr4Mo4V Steel

**DOI:** 10.3390/ma18051092

**Published:** 2025-02-28

**Authors:** Xue Liu, Tao Xia, Hongfei Li, Tianpeng Song, Nan Qu, Yong Liu, Jingchuan Zhu

**Affiliations:** School of Materials Science and Engineering, Harbin Institute of Technology, Harbin 150001, China

**Keywords:** 8Cr4Mo4V steel, metamorphic layer, grinding hardening, physical simulation, stress-induced austenite transformation, nanoindentation behavior

## Abstract

The formation of surface austenite leads to microstructural changes, causing grinding hardening. However, the effect of grinding mechanical stresses on surface austenitization remains unclear. Additionally, the mechanical properties of the metamorphic layer are crucial for studying grinding hardening. Therefore, in this study, the evolution of the microstructure and corresponding mechanical properties of the grinding surface in 8Cr4Mo4V steel was analyzed. The microstructure of the metamorphic layer was characterized using scanning electron microscopy (SEM) and transmission electron microscopy (TEM). Physical simulation was employed to analyze the effect of mechanical compressive stress on the austenite transformation start temperature (Ac1). Dimensionless analysis, based on nanoindentation results, was conducted to study the mechanical properties of the metamorphic layer. The metamorphic layer in 8Cr4Mo4V steel consists of martensite, retained austenite, and undissolved carbides. The unresolved carbides are distributed within the cryptocrystalline martensite. Increasing the grinding depth and workpiece feed speed results in higher mechanical stress and temperature, which leads to a reduction in Ac1 and a higher content of austenite. The yield strength of the metamorphic layer is 2427 MPa, which is 427 MPa higher than that of the matrix, indicating obvious grinding hardening.

## 1. Introduction

8Cr4Mo4V steel, as a second-generation bearing steel [1,2], exhibits excellent mechanical properties and thermal stability, making it suitable for the manufacture of main shaft bearings in aircraft engines [3,4,5,6,7]. The manufacturing process of the bearings includes forging, rough turning, heat treatment, grinding, pickling, and fine grinding [8]. Grinding is a crucial step after turning to enhance the dimensional accuracy and surface quality of the rolling elements, significantly influencing the performance of the bearing products [9,10]. The notable characteristics of grinding are high heat generation, elevated temperatures, and high strain rates [11,12]. The increase in temperature and plastic deformation inevitably lead to changes in the surface microstructure, such as grain refinement and phase transformation [13,14,15]. This microstructure on the ground surface that differs from the bulk material is referred to as the metamorphic layer. Since the failure of 8Cr4Mo4V steel bearings often originates from the delamination of the surface layer [16,17,18], it is particularly important to analyze the microstructure and mechanical properties of the metamorphic layer formed during grinding.

Since it was proposed by Brinksmeier and Brockhoff in 1996 [19], the formation and microstructural distribution of the metamorphic layer have been major focuses in the study of grinding hardening [9,20,21,22,23]. Initially, the metamorphic layer, identified by its dark color, was distinguished from the matrix material (bright) by comparing the colors of the surface and the core [8,9]. Further research revealed that the metamorphic layer is composed of martensite and carbides [24]. The formation of martensite is inherently linked to surface phase transformations. Zarudi [25] studied the grinding of AISI4140 steel and observed that the martensite grains in the deeper regions of the metamorphic layer were coarser. Zhang et al. [26] successfully explained the gradient distribution of grain sizes by using thermocouples to obtain the temperature distribution on the grinding surface. Subsequent researchers confirmed the consistency between the temperature from triangular equivalent heat source numerical simulations and experimental results [27,28]. The surface temperature during grinding and the retained austenite in the metamorphic layer post-cooling provided evidence of surface austenitization [21]. Mao [29] found that, even below the starting temperature of austenite transformation, the metamorphic layer still contained martensite and retained austenite. This indicates that mechanical stress, in addition to temperature, is another significant factor in surface phase transformations. Therefore, the influence of mechanical stresses on surface austenitization remains unclear and needs further investigation.

The grinding surface hardening in grinding is related to the grain refinement and the content of martensite in the metamorphic layer [30]. The plastic deformation of surface grains produces a large number of dislocations. When it accumulates to a certain content, recrystallization occurs [31]. Sun et al. [9] combined finite element analysis with the Cellular Automaton (CA) model to study the mechanism of grain refinement caused by grinding. Nonlinear dynamic thermo-mechanical coupling is the main cause of dynamic recrystallization in austenite. They also found that the thickness of the refined layer does not exceed that of the metamorphic layer. Liu et al. [32] obtained the microhardness distribution of the metamorphic layer in AISI 1060 steel after grinding. Combined with the microstructure distribution, the surface-hardening layer was divided into a fully hardened zone and a transition zone. Guo et al. [24] analyzed the effects of grinding process parameters on the hardening depth of the metamorphic layer, suggesting that increasing the grinding depth and feed rate can increase the hardening depth. Yao et al. [33] investigated the relationship between the microstructure causing surface hardening and grinding forces and temperature, revealing the formation mechanism of the grinding strengthening layer. Analyzing the surface microstructure evolution can effectively help to understand the formation of the surface-hardening layer. However, due to the thickness of the metamorphic layer, there are fewer studies on its mechanical properties. To more clearly analyze the changes in the mechanical properties before and after grinding of 8Cr4Mo4V steel, the dimensionless method, which can obtain single-phase deformation constitutive relationships [34,35,36,37], is used for research.

Investigating the microstructure and mechanical properties of the grinding metamorphic layer in 8Cr4Mo4V steel is of significant importance for optimizing grinding process parameters. Additionally, further analysis is required to understand the impact of grinding mechanical stresses on the phase transformation of the ground surface. Therefore, in this study, SEM and TEM were employed to characterize the microstructure of the metamorphic layer in 8Cr4Mo4V steel, revealing its microstructure. By changing the grinding process parameters, the effects of grinding forces and heat on the thickness of the metamorphic layer and the content of residual austenite are analyzed. Through physical simulation experiments, the influence of grinding mechanical stresses on the phase transformation behavior of 8Cr4Mo4V steel is revealed. To explore the mechanical properties of the metamorphic layer, nanoindentation tests were conducted. The test results were subjected to dimensionless analysis to derive the deformation constitutive relationship of the metamorphic layer. Analyzing the evolution of the microstructure and the mechanical properties of the metamorphic layer is crucial for adjusting bearing grinding processes, effectively controlling the microstructure of the metamorphic layer, and achieving surface grinding hardening.

## 2. Materials and Methods

### 2.1. Materials and Heat Treatment

The chemical composition of the 8Cr4Mo4V steel is listed in Table 1. The initial state of 8Cr4Mo4V steel was annealed. The samples were heated to 1090 °C and held at this temperature for 30 min. Subsequently, high-pressure nitrogen was used for quenching. After quenching, the samples were immediately subjected to tempering. The samples were heated to 550 °C and held for 150 min. This tempering process was repeated three times to minimize the residual austenite. After the heat treatment, wire cutting was used to machine the tensile and grinding samples.

### 2.2. Grinding Experiment and Physical Simulation

#### 2.2.1. Process Parameters of Grinding Experiment

Surface grinding was performed using a white corundum grinding wheel. The grinding wheel’s grit specification, outer diameter, inner diameter, and width were 100#, 200 mm, 20 mm, and 20 mm, respectively. The line speed of the grinding wheel was 10 m/s. To investigate the microstructure of the metamorphic layer in 8Cr4Mo4V steel after grinding at different grinding depths and feed speeds, the grinding process parameters were designed and listed in Table 2. The grinding method employed was surface grinding, and the cooling method was air cooling.

#### 2.2.2. Process Parameters of Physical Simulation

To analyze the microstructural evolution of 8Cr4Mo4V steel during grinding, physical simulations were conducted using a Gleeble 1500D dynamic thermomechanical simulator (Produced by DSI in USA). The cylindrical specimens used had a diameter of 8 mm and a length of 12 mm. Before the physical simulation, the oxidized layer on the surface of the cylindrical specimens was removed using sandpaper. During the simulation, compressive stresses of 50 MPa, 80 MPa, 100 MPa, and 120 MPa were applied to both ends of the specimens. The specimens were then heated at a rate of 10 °C/s to 900 °C and held at this temperature for 2 s before being air-cooled to room temperature. During the experiment, a dilatometer was used to measure the diameter changes in the middle section of the cylindrical specimens. The phase transformation process of 8Cr4Mo4V steel under thermomechanical coupling was analyzed through the diameter change curves of the specimens.

### 2.3. Microstructure Characterization and Mechanical Property Test

#### 2.3.1. Microstructure Analysis

The microstructural morphology of the metamorphic layer after polishing and etching was observed using an Aziotech optical microscope (Produced by Zeiss) and field emission Merlin Compact scanning electron microscope (Produced by Zeiss) equipped with an Energy Dispersive Spectrometer (EDS). The grinding surface was etched with a mixture of saturated picric acid (100 mL) and hydrochloric acid (10 mL) to analyze the grain size changes in the metamorphic layer. A mixture of nitric acid (5 mL) and alcohol (95 mL) was used to reveal the microstructure of the metamorphic layer. The microstructure of the metamorphic layer was characterized using a Tecnai G2 F20 transmission electron microscope (Produced by FEI) after ion thinning. The phase crystal structure and content of the 8Cr4Mo4V steel bearing were analyzed using the Empyrean X-ray diffractometer (XRD) equipped with CuKa radiation using the scanning angles from 30° to 120°, an interval step of 0.02°, a voltage of 40 kV, and a current of 30 mA. The formula for calculating the content of phases in the grinding surface is as follows:

(1) $$V_{\gamma }=1/\left(1+R_{\gamma }I_{\alpha }/R_{\alpha }I_{\gamma }\right),$$

where $I_{\alpha }$ and $I_{\gamma }$ are the integrated intensities of diffraction peaks in the bcc phase and fcc phase, respectively. $V_{\gamma }$ is the volume fraction of the residual austenite; $R_{\alpha }$ and $R_{\gamma }$ depend on *θ*, (hkl), and the kind of substance.

#### 2.3.2. Room-Temperature Tensile Test

**Figure 1:**
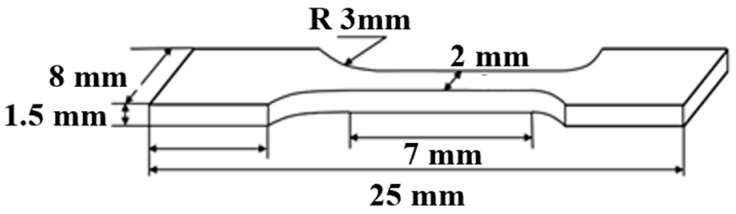
The dimensions of a tensile specimen.

The influence of grinding on the mechanical properties of 8Cr4Mo4V steel was revealed through room-temperature tensile testing. The tests were conducted using a universal mechanical testing machine with a maximum load capacity of 250 kN, at a tensile speed of 0.2 mm/s. The dimensions of the tensile specimens are shown in Figure 1

#### 2.3.3. Nanoindentation Test and Numerical Simulation

Nanoindentation testing provided insights into the mechanical properties of the metamorphic layer. Before the test, the oxidized surface of the metamorphic layer was removed with a mixture of nitric acid and alcohol to reveal the microstructure. To ensure the reproducibility and accuracy of the nanoindentation test results, three repeated tests were performed on the surface of the specimen. The nanoindentation tests were carried out using a G200 nanoindentation instrument with a maximum indentation depth set at 1200 nm.

**Figure 2:**
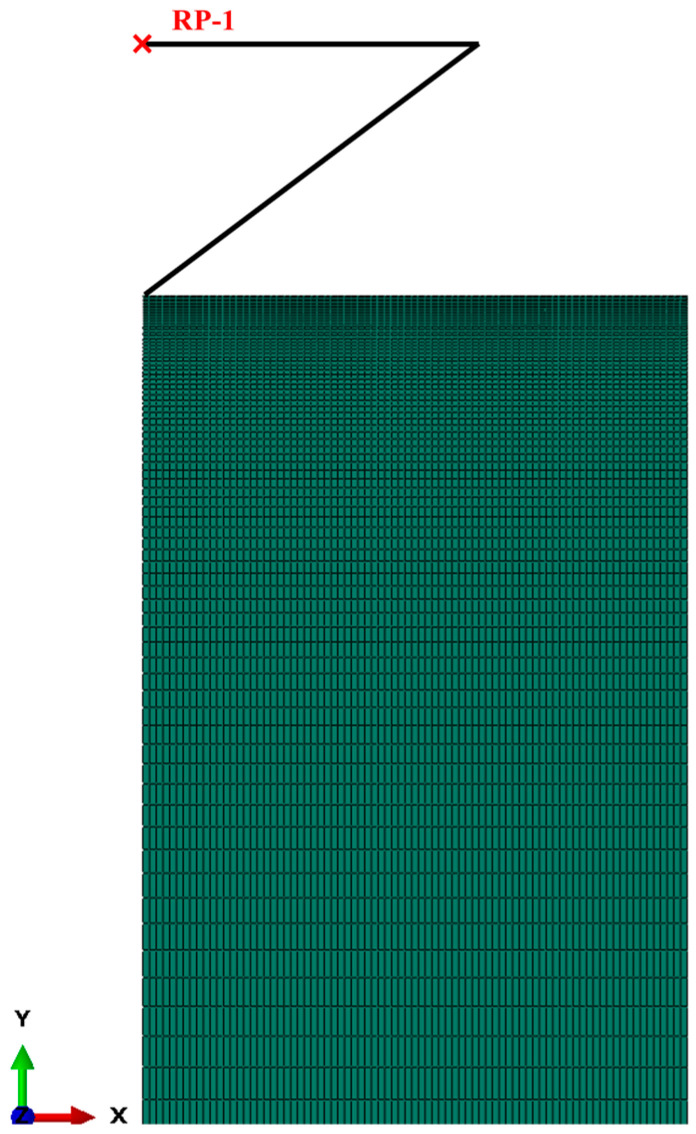
Nanoindentation model and grid mesh results.

In the dimensionless analysis of the mechanical properties of the metamorphic layer, it is necessary to determine the representative plastic strain. This critical parameter is obtained through a self-consistent iterative finite element numerical simulation. The two-dimensional nanoindentation process is simulated in the ABAQUS 2020 finite element software. The nanoindenter uses Berkovich indenter, which is approximated as a conical shape. The two-dimensional plane model is a triangle with an angle of 70.3°. The indenter is set as a discrete rigid body to ignore its deformation during indentation. Due to the significant surface deformation of the model, the surface grid needs to be meshed more finely to improve the accuracy of the simulation results. The meshing results of the model are shown in Figure 2. The X-axis symmetry constraints are applied on the left-hand side of the model, and full fixation constraints in the Y direction are applied at the bottom. The movement of the indenter is controlled by applying the displacement in the Y direction to simulate the loading and unloading process of nanoindentation. It is also necessary to define the type of interaction between the indenter and the model during this process. The surface of the indenter is set as the first surface, and the upper surface of the model is set as the second surface. The loading stage and the unloading stage are set as two analysis steps, and the size of the computational increment step is automatically adjusted based on the convergence situation.

## 3. Results

### 3.1. Microstructure of Metamorphic Layer

**Figure 3:**
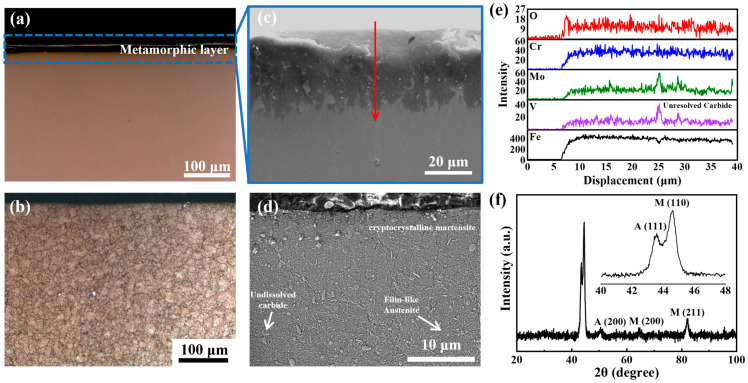
Microstructure of surface metamorphic layers with a grinding depth of 10 μm and a feed speed of 40 mm/s. (**a**) Non-corrosive surface metallography after polishing, in which the dark layer is a metamorphic layer; (**b**) the distribution of grain boundary in the metamorphic layer after etching, showing the grain size distribution along the gradient; (**c**) SEM image of metamorphic layers without etching; (**d**) SEM image of the microstructure of the etched metamorphic layer, which shows the distribution of the phases; (**e**) the analysis result of the element content along the red arow; (**f**) the XRD spectrum of the metamorphic layer.

After the surface grinding of the 8Cr4Mo4V steel, the microstructure morphology of the cross-section is shown in Figure 3, where the surface metamorphic layer is clearly visible. Figure 3a presents a metallographic image of the sample in an unetched state after grinding. The dark layer represents the metamorphic layer, and the gray layer indicates the tempered martensitic of the matrix. The thickness of the dark layer is approximately 30 μm in Figure 3b. When observing the grinding surface using SEM, the differences in scattering and reflection capabilities of the incident electron beam between the metamorphic layer and the matrix result in variations in the number of secondary electrons emitted. This leads to the appearance of a dark layer and a gray layer in the SEM images. Figure 3b,d show the surface morphology after etching. Figure 3b shows that the grain size from the metamorphic layer to the substrate is distributed in a gradient along the thickness direction. This gradient is associated with the temperature, stress, and strain field gradients present at the surface during the grinding process. The gradient microstructure effectively improves surface hardness. The metamorphic layer primarily consists of martensite, retained austenite, and undissolved carbides. The martensitic morphology in the metamorphic layer indicates the presence of cryptocrystalline martensite. A small amount of retained austenite and undissolved carbides are distributed within the martensite.

Under the combined effects of grinding heat and grinding force, the surface temperature exceeds the Ac1, causing the tempered martensite to transform into austenite [21]. The nonlinear dynamic thermo-mechanical coupling at the grinding surface leads to dynamic recrystallization of the surface austenite [9]. Since the surface temperature and stress exhibit gradient distributions, the surface deformation grain and recrystallization grain also show gradient distributions along the thickness direction. After cooling, the austenite at the grinding sample surface transforms into cryptocrystalline martensite [38], while some untransformed austenite remains as retained austenite. During the surface austenitizing process, carbide in tempered martensite gradually dissolves into austenite. However, due to the relatively high grinding wheel speed and short contact time with the workpiece surface, incomplete dissolution of carbides occurs.

To provide a clearer understanding of the composition of the metamorphic layer, the variations in the elemental content of the grinding surface were analyzed, as shown in Figure 3c. The oxygen content on the surface of the metamorphic layer is high, and the distribution is a gradient (Figure 3e). However, diffraction peaks of corresponding oxides are not found in XRD (Figure 3f). Therefore, the distribution of oxygen elements is related to the oxygen diffusion of the surface during grinding. The undissolved carbides in the metamorphic layer are identified as alloy carbides, with the primary alloying elements being V and Mo. The XRD results of the grinding metamorphic layer are presented in Figure 3f. The magnification in Figure 3f shows the (110) diffraction peak of martensite and the (111) diffraction peak of residual austenite. In addition, the (200) diffraction peak of residual austenite is also obvious. Therefore, there is residual austenite in the grinding metamorphic layer.

**Figure 4:**
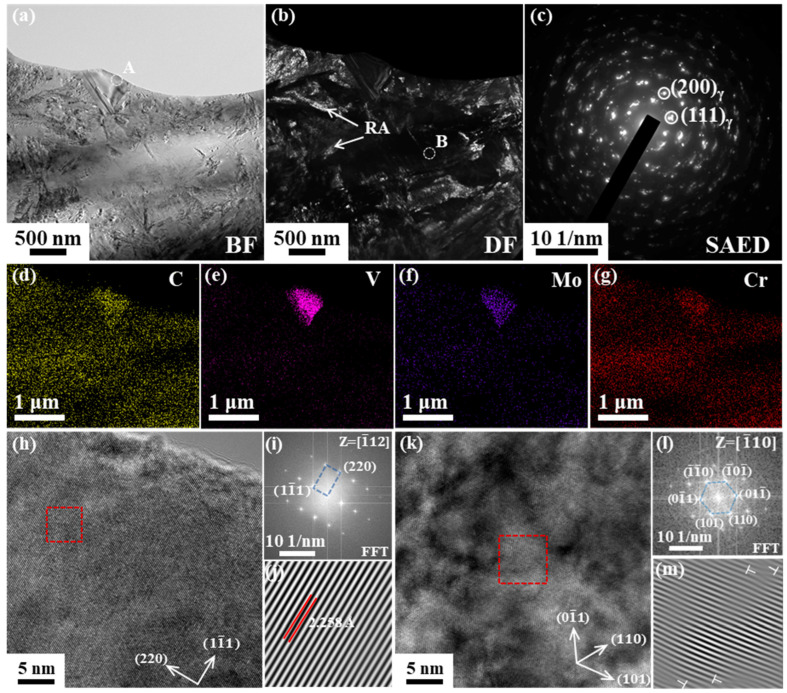
TEM characterization results of grinding metamorphic layer with a grinding depth of 10 μm and a feed speed of 40 mm/s. (**a**) Microscopic morphology of metamorphic layer (bright field); (**b**) the dark field microscopic morphology of metamorphic layer; (**c**) the selective electron diffraction spots of retained austenite at position B in (**b**); (**d**–**g**) are the distribution of elements C, V, Mo, and Cr in metamorphic layer, corresponding to the position A of (**a**); (**h**,**k**) are the HRTEM characterization results of undissolved carbide and martensite; (**i**,**l**) are the diffraction spots of undissolved carbide and martensite through Fast Fourier Transformation (FFT) of the red square region in (**h**,**k**); (**j**,**m**) are the results of IFFT.

After removing the oxide layer from the metamorphic layer’s surface, the TEM characterization results of the metamorphic layer are shown in Figure 4. Figure 4a displays the microstructure morphology within a 2 μm thickness of the metamorphic layer surface. After the heat treatment, there are two types of martensite in 8Cr4Mo4V steel: twinned martensite and lath martensite. However, due to the effect of grinding heat and force, cryptocrystalline martensite is produced in the metamorphic layer, leading to changes in the martensite’s morphology. The dark field image of the surface microstructure is shown in Figure 4b. The retained austenite in the metamorphic layer exhibits block-like and film-like shapes, consistent with the morphology of retained austenite in 8Cr4Mo4V steel before grinding [3]. Figure 4c shows the selected-area electron diffraction (SAED) result of the retained austenite. Diffraction spots indicate that the residual austenite grains are refined. Severe plastic deformation occurs on the ground surface, resulting in an increase in dislocation density. When the density of dislocation increases to meet the requirements of dynamic recrystallization, dynamic recrystallization occurs, which leads to the refinement of austenite grains to the nanoscale. Based on the elemental distribution (Figure 4d–g), it is found that undissolved carbides in the metamorphic layer primarily contain alloy elements V and Mo. According to research [3], the types of carbides in 8Cr4Mo4V steel are mainly M2C and MC. The HRTEM characterization results and the diffraction spots suggest that the undissolved carbide type is M2C. By performing the Inverse Fast Fourier Transformation (IFFT), it is observed that no dislocations are generated in the undissolved carbides after grinding. HRTEM results of martensite in the metamorphic layer reveal the presence of a large number of dislocations. The combination of martensite and nanoscale retained austenite in the metamorphic layer contributes to the hardening of the grinding surface.

### 3.2. Effect of Grinding Parameters on Metamorphic Layer

#### 3.2.1. Grinding Depth

**Figure 5:**
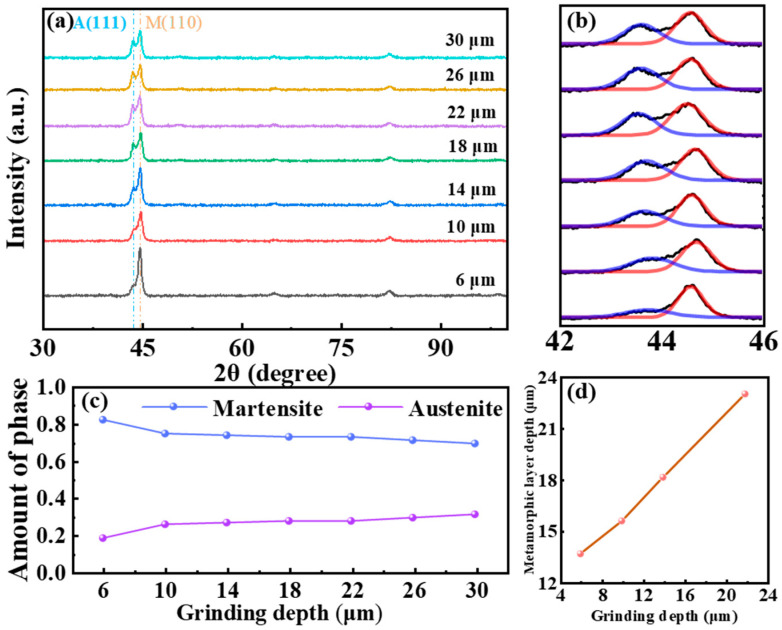
XRD analysis results and thickness of metamorphic layer at 40 mm/s (feed speed) and different grinding depths. (**a**) XRD maps of different grinding depths; (**b**) peak-fitting results of XRD graph (The blue represents the A(111) and the red represents the M(100)); (**c**) changes in the content of martensite and austenite in the metamorphic layer; (**d**) the thickness of the metamorphic layer corresponding to different grinding depths.

Figure 5 shows the effect of grinding depth on the phase content and thickness of the metamorphic layer. With the grinding depth increasing, the intensity of the retained austenite diffraction peaks in Figure 5a gradually increases, indicating an increase in the retained austenite content in the metamorphic layer after the grinding. According to the Chinese industry standard YB/T 5338–2006, the (220) and (211) diffraction peaks of martensite and the (200), (220), and (311) diffraction peaks of austenite are selected for calculation. The calculation results from the combination of these diffraction peaks are averaged. Calculating the retained austenite content using multiple combinations of diffraction peaks can improve the accuracy of the calculation results. However, the intensities of other diffraction peaks are extremely low, excepting the strongest peaks of martensite and retained austenite. To quantitatively analyze the content of martensite and retained austenite in the metamorphic layer, peak fitting was performed on the (110) diffraction peak of martensite and the (111) diffraction peak of austenite to obtain their respective diffraction peak intensities. The fitting results are shown in Figure 5b, with the fitting accuracy of each diffraction peak being over 95%. Figure 5c shows the changes in the content of martensite and retained austenite calculated using Equation (1). The content of martensite in the metamorphic layer gradually decreases, while the content of retained austenite increases. This is related to the area of the grinding arc. Larger grinding depth results in a larger contact area between the grinding grain and the surface, leading to increased grinding temperature and mechanical stress. This results in a higher degree of surface austenitization, more dissolved carbides, and an increase in carbon concentration in the austenite, which lowers the martensite transformation temperature during the cooling and increases the content of retained austenite. The changes in the thickness of the metamorphic layer due to the variations in grinding heat and stress caused by the increase in grinding depth are shown in Figure 5d. As the grinding depth increases from 6 μm to 22 μm, the thickness of the metamorphic layer increases, which is consistent with the results of Zhang [8].

#### 3.2.2. Grinding Feed Speed

**Figure 6:**
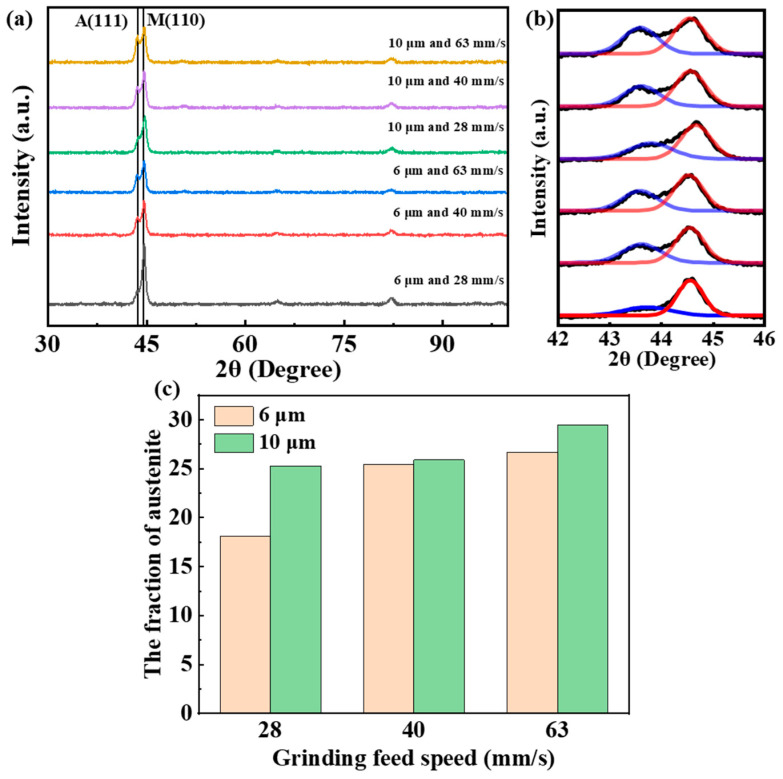
Effect of different grinding feed speeds on the phase composition in the metamorphic layer. (**a**) XRD patterns corresponding to various grinding parameters; (**b**) peak-fitting results for the martensite (110) diffraction peak and the austenite (111) diffraction peak (The blue represents the A(111) and the red represents the M(100)); (**c**) austenite content in the metamorphic layer.

Figure 6 shows the effect of feed speed on the phase composition of the metamorphic layer. With the increase in workpiece feed speed, the intensity of the austenite diffraction peak increases, indicating an increase in austenite content in the grinding metamorphic layer. The austenite content, calculated based on peak-fitting results, is shown in Figure 6c. For different feed speeds, the austenite content at a 10 μm grinding depth is higher than that at a 6 μm grinding depth. At the same grinding depth, as the feed speed increases from 28 mm/s to 63 mm/s, the austenite content reaches 29.5%. With an increase in grinding feed speed, more material needs to be removed per unit of time, requiring more energy to be transferred to the workpiece surface, leading to an increase in surface temperature and, consequently, higher austenite content on the surface.

### 3.3. Mechanical Properties of Metamorphic Layer

The microstructural transformation on the grinding surface inevitably results in different mechanical properties in the metamorphic layer compared to the matrix material. In this section, the nanoindentation method is used to study the mechanical properties of the grinding metamorphic layer in 8Cr4Mo4V steel. The influence of the metamorphic layer on the mechanical properties of 8Cr4Mo4V steel is analyzed by examining the fracture morphology of the metamorphic layer under room-temperature tensile testing.

#### 3.3.1. Nanoindentation Behavior Analysis

**Figure 7:**
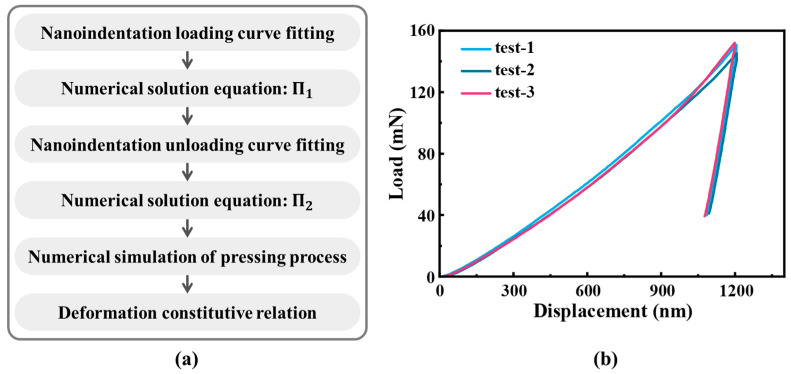
(**a**) Flowchart for analyzing the mechanical properties of metamorphic layers by a dimensionless method; (**b**) after grinding with the grinding depth of 10 μm and feed speed of 40 mm/s, the load–displacement curves of the metamorphic layer were obtained through three nanoindentation tests.

The grinding metamorphic layer has a thickness of approximately several tens of microns, making it challenging to conduct mechanical performance tests. The dimensionless method, which uses a tiny indenter to obtain the load–displacement curves, has become an effective approach for analyzing the mechanical properties of micro-regions. The dimensionless method was initially proposed by Dao et al. [34] to obtain the stress–strain curves of materials from nanoindentation load–displacement curves. The general, dimensionless functions were constructed to characterize the sharp indentation of the instrument. Based on these functions and elastoplastic finite element calculations, the indentation data were linked with the elastoplastic data. The deformation constitutive relationship can be obtained from the nanoindentation data (reverse analysis), and the key parameters of nanoindentation can also be obtained from the constitutive relationship (forward analysis). Since the thickness of the metamorphic layer is only a few dozen micrometers, it is difficult for common testing methods to obtain elastoplastic deformation data. Therefore, the load–displacement curves of the metamorphic layer were obtained through nanoindentation testing. Based on the dimensionless method, the representative plastic stress, representative plastic strain, and strain-hardening index of the metamorphic layer were obtained from the load–displacement curves of the nanoindentation, and the deformation constitutive relationship was constructed. The corresponding analysis process is presented in Figure 7a.

When the Berkovich indenter presses into the metamorphic layer, the indentation load *P* is related to the indentation depth *h*, the elastic modulus *E* of the metamorphic layer, the elastic modulus *E*_i_ of the indenter, the yield strength *σ*_y_ of the metamorphic layer, and the strain-hardening index *n*. The indentation load *P* can be expressed as follows:

(2) $$P=P(h,E,v_{i},E_{i},\sigma_{y},n).$$

Using the effective elastic modulus *E*_r_ to replace the elastic modulus of the indenter and the metamorphic layer, the indenter load is rewritten as Equation (4). The form of dimensionless function Π_1_ can be found in Appendix A.

(3) $$E_{r}=\frac{1}{\left(\frac{1-\nu^{2}}{E}+\frac{1-\nu_{i}^{2}}{E_{i}}\right)},$$

(4) $$P=\sigma_{r}h^{2}\Pi_{1}(\frac{E_{r}}{\sigma_{r}},n),$$

where $\sigma_{r}$ is the representative plastic stress, which is a key parameter in constructing the deformation constitutive relationship of the metamorphic layer. The load–displacement curve of the nanoindentation is shown in Figure 7b, with small deviations between the three test results, and the modulus and hardness of the metamorphic layer obtained are listed in Table 3. A dimensionless analysis is performed using the results of the first test, and the loading curve is fitted with $P=Ch^{2}$ to obtain the constant *C*. Combining the Π_1_ equation obtained by Dao et al. [34], the characteristic plastic stress of the metamorphic layer is numerically solved. For the unloading curve, $P=A{\left(h-h_{f}\right)}^{m}$ is used, where *h*_f_ is the residual indentation depth, to fit the constants *A* and the exponent *m*. Equation (5) is the dimensionless equation for the load during unloading.

(5) $$P_{u}=E_{r}h^{2}\Pi_{2}(\frac{h_{m}}{h},\frac{\sigma_{r}}{E_{r}},n),$$

According to the Π_1_ equation in the reference [34], the strain-hardening index is numerically obtained. The elastoplastic constitutive relationship of metallic materials can be written using a power-law function as follows:

(6) $$\sigma =\left\{\begin{array}{cc} E\varepsilon & \sigma \leq \sigma_{y}\\ \sigma_{y}{\left(1+\frac{E}{\sigma_{y}}\varepsilon_{p}\right)}^{n} & \sigma >\sigma_{y} \end{array}\right.$$

Currently, the representative plastic stress and strain-hardening index have been obtained. To construct Equation (6), it is necessary to obtain the representative plastic strain of the metamorphic layer to determine the yield strength. The representative plastic strain of the metamorphic layer is obtained using forward analysis. A nanoindentation model is built using Abaqus, incorporating *σ*_r_, *n*, and the initialized *ε*_r_ to construct the constitutive relationship of the metamorphic layer. The nanoindentation process is simulated to obtain the simulated load–displacement curve. The *ε*_r_ of the metamorphic layer is obtained using a self-consistent iterative method. The iteration converges when the difference between the maximum load obtained from the simulation and the maximum load on the load–displacement curve does not exceed 1%. The relationship between $\varepsilon_{r}^{n+1}$ and $\varepsilon_{r}^{n}$ for the next iteration is as follows:

(7) $$\varepsilon_{r}^{n+1}=\varepsilon_{r}^{n}\frac{P_{\exp}}{P_{\mathrm{sim}}}.$$

Through the backward analysis using the dimensionless method, the characteristic plastic stress, characteristic plastic strain, and strain-hardening index of the metamorphic layer have been obtained. These key parameters obtained throughout the analysis process are listed in Table 4.

The results of the dimensionless analysis are consistent with the yield strength of the metamorphic layer obtained using the crystal plasticity finite element method in the literature [11], which ranges from 2000 MPa to 2500 MPa. The yield strength of the tempered martensite for 8Cr4Mo4V steel using the same method was obtained as 2000 MPa, and the strain-hardening index was 0.46. After the grinding, the yield strength of the metamorphic layer increased by 427 MPa, and the strain-hardening index decreased by 0.04. The increase in yield strength of the metamorphic layer is related to its microstructure. Grinding leads to grain refinement on the surface, according to the Hall–Petch relationship, which enhances the yield strength. The metamorphic layer contains cryptocrystalline martensite after the cooling, which also improves the surface strength. Although there is a significant amount of retained austenite in the metamorphic layer after the grinding, it undergoes intense plastic deformation under mechanical stress, leading to the formation of nanograins. The surface hardness can be enhanced by the presence of large deformed grains in the outer layer of the metamorphic layer after grinding.

#### 3.3.2. Effect of Metamorphic Layer on Tensile Fracture Behavior

**Figure 8:**
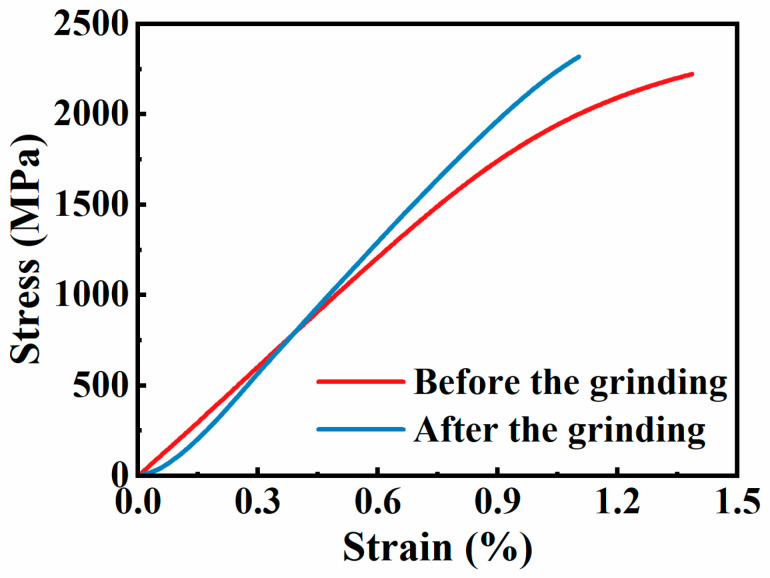
Stress–strain curve of room-temperature tensile before and after the grinding.

Figure 8 shows the room-temperature tensile stress–strain curves of the tensile specimens before and after the grinding. Both tensile curves exhibit the fracture characteristics of brittle material. After the grinding, the fracture strength of the specimen increased from 2235 MPa to 2320 MPa, while the elongation decreased.

**Figure 9:**
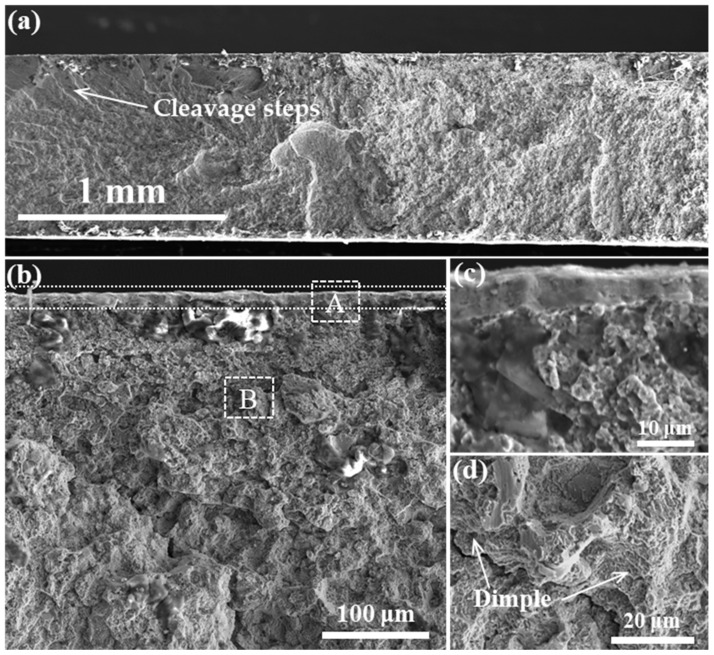
Fracture morphology of the specimen after grinding with the grinding depth of 10 μm and feed speed of 40 mm/s. (**a**,**b**) show SEM images of the fracture surface at different magnifications; (**c**) The morphologies of the metamorphic layer on the fracture plane at position A in (**b**) are enlarged, showing typical brittle fracture characteristics; (**d**) The morphology of the matrix on the fracture plane at position B in (**b**) is enlarged, showing quasi-cleavage fracture characteristics.

Figure 9 is the SEM image of the cross-section of the tensile fracture surface of the specimen after the grinding. Figure 9a indicates that no necking occurred during the tensile process, and the presence of cleavage fracture steps on the fracture surface suggests that the crack was initiated in the center of the specimen. The microstructure of the matrix on the tensile fracture surface is shown in Figure 9c. Due to the presence of distinct cleavage fracture steps and dimples, the matrix exhibits a quasi-cleavage fracture. The morphology of the metamorphic layer on the fracture surface is significantly different from that of the matrix, exhibiting typical brittle fracture characteristics.

In the central region of the fracture, numerous dimples are present, indicating that the crack initiation occurred in this area. During crack propagation from the center to the surface, multiple cleavage steps are observed. Finally, the metamorphic layer undergoes shear failure due to the high shear stresses it experiences. The formation of the metamorphic layer does not alter the crack propagation behavior; however, the microstructural changes caused by grinding lead to a higher strength in the metamorphic layer compared to the central region, resulting in an increase in the tensile fracture strength. The extended exposure to grinding heat causes prolonged tempering of the matrix, leading to a decrease in elongation.

## 4. Discussion

### 4.1. Austenization of Grinding Surfaces

Under the combined effects of grinding heat flow and mechanical stress, the tempered martensite on the bearing surface transforms into austenite, leading to changes in the microstructure of the surface after grinding. Numerous studies have been conducted on the effect of heating rates on the formation temperature of austenite [39,40]. However, the influence of mechanical stress on austenitization remains unclear. To more clearly analyze the formation of austenite on the surface, physical simulation was utilized to explore the influence of mechanical stresses on the thermodynamics of austenite transformation for 8Cr4Mo4V steel in this section.

**Figure 10:**
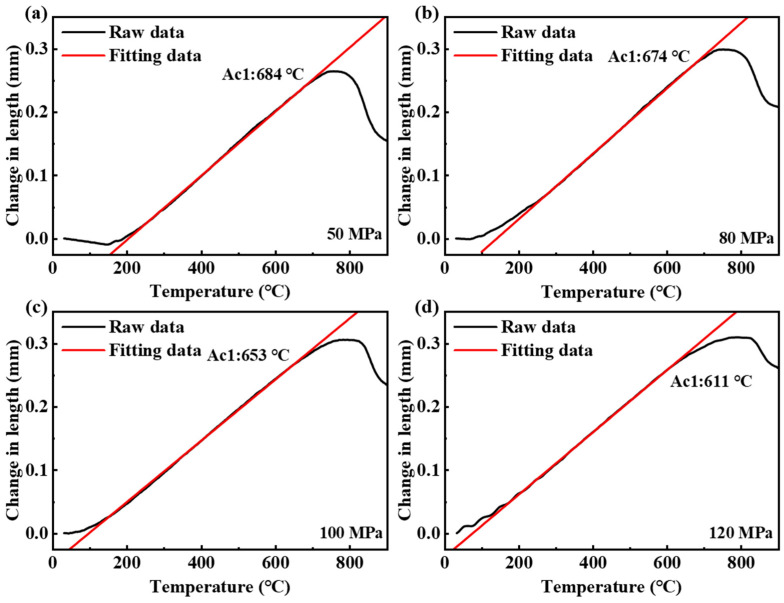
The sample length variation curve in physical simulation process. (**a**–**d**) represent the applied compressive stress values of 50 MPa, 80 MPa, 100 MPa, and 120 MPa, respectively.

The austenitizing temperature for the physical simulation was set at 900 °C, with a heating rate of 10 °C/s and applied compressive stresses of 50 MPa, 80 MPa, 100 MPa, and 120 MPa. The critical temperature for austenite formation was determined by changes in the slope of the dilatation curve. During the heating, the dynamic dilatometer recorded the change in sample diameter with temperature, as shown in Figure 10. Below Ac1, the change in sample length was linearly related to temperature. Once the austenite transformation began, the dilatation curve deviated from linearity due to volume contraction. When the temperature exceeded the critical temperature, austenitization was complete, and the dilatation curve returned to linearity. The austenite transformation was completed between the Ac1 and Ac3 temperatures. Table 5 lists the critical temperatures for the Ac1 obtained using the tangent method under different mechanical stresses. The application of external mechanical compressive stress resulted in a decrease in the austenite formation temperature.

The change in the critical temperature Ac1 for the transformation of tempered martensite to austenite can be explained using the Clausius–Clapeyron theory.

(8) $$\frac{dT_{\alpha \to \gamma }}{\mathrm{dp}}=\frac{∆V_{\alpha \to \gamma }}{∆S_{\alpha \to \gamma }}=\frac{∆V_{\alpha \to \gamma }T_{\alpha \to \gamma }}{∆H_{\alpha \to \gamma }}$$

In 8Cr4Mo4V steel, the lattice constant of the body-centered cubic structure is larger than that of the face-centered cubic structure. During the formation of austenite, the volume expands. So, $∆V_{\alpha \to \gamma }$ is negative. The transformation from tempered martensite to austenite is endothermic, so $∆H_{\alpha \to \gamma }$ is positive. Therefore, during the austenitization of the ground surface, $\frac{dT_{\alpha \to \gamma }}{\mathrm{dp}}<0$. Under the influence of mechanical stresses during grinding, the temperature required for the transformation from tempered martensite to austenite decreases. The austenite transition kinetics of AISI 1045 steel were calculated by Shichao Xiu [21]. The austenite transition temperatures Ac1 and Ac3 decreased under 500 Mpa compressive stress. The results of physical simulation and calculation indicate the austenization of the grinding surface.

The larger the applied mechanical compressive stress, the lower the critical temperature for the phase transformation. The effect of pressure stress on the austenite transition temperature can also be explained by phase transition thermodynamics. The decrease in Ac1 is related to the reduction in the free energy barrier for austenitization. Similar to stress-induced martensitic transformation, the deformation energy generated by the applied mechanical stress can act as a driving force for the phase transformation, reducing the required chemical free energy. Additionally, under compressive stress, the volume contraction during austenitization is facilitated.

During the grinding process, the temperature and mechanical stress on the sample surface are closely related to the grinding depth and the workpiece feed rate. As the grinding depth increases, the arc area of contact between the grinding wheel and the sample surface enlarges, leading to an increase in the grinding temperature. Simultaneously, since more material needs to be removed, the mechanical stress exerted by the grinding wheel on the sample surface also increases, thereby reducing Ac1 on the grinding surface. It is noteworthy that the effects of grinding depth on surface temperature and Ac1 are opposite, which leads to an increase in both the thickness and degree of surface austenitization. Therefore, the decrease in Ac1 and the increase in the degree of austenitization caused by the increase in grinding depth are the main reasons for the increased content of retained austenite in the metamorphic layer.

### 4.2. Microstructure Evolution of Grinding Surface

Under the influence of grinding heat and surface mechanical stress, the transition temperature of tempered martensite to austenite decreases, resulting in the austenitization of the workpiece surface. A large deformation of surface austenite results in an increase in dislocation density in austenite [9]. There is a critical dislocation density for dynamic recrystallization at the current grinding temperature [41]. When the dislocation density increases to a certain extent, the nonlinear dynamic thermo-mechanical coupling results in the dynamic recrystallization of large deformed austenite [9]. The recrystallized grains nucleate in the region of high dislocation density. Due to the short grinding mechanical stress and grinding heat action, the grains of residual austenite after recrystallizing did not coarsen. Although the residual austenite content in the metamorphic layer is high, grinding hardening can still occur because it is refined to the nanometer level. In the grinding process, the grinding wheel speed is high, and the feed speed is large so that the contact time between the grinding wheel and the surface is short. As a result, some carbides in the tempered structure were not dissolved. In addition, during the cooling quenching process, austenite is transformed into cryptocrystalline martensite [38]. The untransformed austenite is retained in martensite in film-like [3,42] and bulk form [3]. The grinding temperature can only exceed Ac1 within a certain thickness range of the grinding surface. The farther away from the surface, the lower the degree of austenization and the higher the tempered martensite content. According to Zhang [8], this is the mixed layer. In the surface region below Ac1, tempered martensite does not undergo an austenitic transformation, but the grain size and distribution of carbides are affected due to grinding heat and mechanical stress. Therefore, the large plastic deformation, caused by mechanical stress, increases the surface defects and the refinement of austenite grains, thus effectively improving the surface strength.

## 5. Conclusions

By characterizing the microstructure of the grinding metamorphic layer in 8Cr4Mo4V steel, the evolution process of the metamorphic layer’s microstructure was analyzed. Physical simulations were conducted to analyze the effects of thermo-mechanical coupling on austenite transformation in 8Cr4Mo4V steel to reveal the austenitization of the grinding surface. Based on nanoindentation curves, dimensionless analysis was employed to establish the deformation constitutive relationship of the metamorphic layer. Room-temperature tensile tests were performed to analyze the impact of the affected layer on mechanical properties. The main conclusions obtained in this article are as follows:

(1) The microstructure of the grinding metamorphic layer in 8Cr4Mo4V steel consists of martensite, retained austenite, and undissolved carbides. The surface of a metamorphic zone is composed of large deformation grains and recrystallized grains.
(2) Increasing the grinding depth and workpiece feed speed results in higher grinding temperature and mechanical stress and leads to an increase in the thickness of the metamorphic layer and the content of austenite.
(3) The grinding mechanical compressive stress can lower the start temperature of austenite transformation in 8Cr4Mo4V steel. With the mechanical compressive stress increasing, the Ac1 decreases, and the content of austenite increases.
(4) The dimensionless analysis based on nanoindentation indicates that the yield strength of the metamorphic layer is 2427 MPa, which is 427 MPa higher than the matrix material. The metamorphic layer enhances the tensile fracture strength, and its fracture characteristics exhibit a brittle fracture.

## Figures and Tables

**Table 1:** Chemical composition of the 8Cr4Mo4V steel (wt.%).

C	Cr	Mo	V	Mn	Fe
0.8	4.0	4.0	1.0	0.2	Bal.

**Table 2:** Process parameters for grinding workpiece.

No. Workpiece	Grinding Depth (μm)	Feeding Speed (mm/s)
1	6	40
2	10	40
3	14	40
4	18	40
5	22	40
6	26	40
7	30	40
8	6	28
9	6	63
10	10	28
11	10	63

**Table 3:** Modulus and hardness of metamorphic layers by nanoindentation test.

Parameters	Test-1	Test-2	Test-3	Average
Young’s modulus (GPa)	224	213.3	220.1	219.1
Hardness (GPa)	9.2	8.9	8.9	9.0

**Table 4:** Key parameter values obtained by dimensionless analysis.

C	A	m	*σ*_r_ (MPa)	*n*	$\varepsilon_{r}$	*σ*_y_ (MPa)
111.96	0.192	1.279	2917	0.42	0.0292	2427

**Table 5:** The Ac1 under different compressive stresses.

Compressive Stress (MPa)	0	50	80	100	120
Ac1 (°C)	821 [3]	684	674	653	611

## Data Availability

The original contributions presented in this study are included in the article. Further inquiries can be directed to the corresponding authors.

## Appendix A

In Section 3.3.1 (Nanoindentation Behavior Analysis), the specific forms of the dimensionless equations Π_1_ and Π_2_ are as follows:

(A1) $$\Pi_{1}=\frac{C}{\sigma_{r}}=-1.131{\left[\ln\left(\frac{E_{r}}{\sigma_{r}}\right)\right]}^{3}+13.635{\left[\ln\left(\frac{E_{r}}{\sigma_{r}}\right)\right]}^{2}-30.594\left[\ln\left(\frac{E_{r}}{\sigma_{r}}\right)\right]+29.267$$

(A2) $$\begin{array}{ll} \Pi_{2}= & \frac{1}{E_{r}h_{m}}\cdot \frac{dP_{u}}{dh_{m}}=\left(-1.40557n^{3}+0.77526n^{2}+0.1583n-0.06831\right){\left[\ln\left(\frac{E_{r}}{\sigma_{r}}\right)\right]}^{3}\\ & \;+\left(17.93006n^{3}-9.22091n^{2}-2.37733n+0.86295\right){\left[\ln\left(\frac{E_{r}}{\sigma_{r}}\right)\right]}^{2}\\ & +\left(-79.99715n^{3}+40.55620n^{2}+9.00157n-2.54543\right)\left[\ln\left(\frac{E_{r}}{\sigma_{r}}\right)\right]\\ & \;+\left(122.65069n^{3}-63.88418n^{2}-9.58536n+6.20045\right) \end{array}$$
